# Understanding the Biosynthesis of Paxisterol in Lichen-Derived *Penicillium aurantiacobrunneum* for Production of Fluorinated Derivatives

**DOI:** 10.3390/molecules27051641

**Published:** 2022-03-02

**Authors:** Yoshi Yamano, Harinantenaina L. Rakotondraibe

**Affiliations:** 1 Division of Medicinal Chemistry and Pharmacognosy, College of Pharmacy, The Ohio State University, Columbus, OH 43210, USA; yamano@hiroshima-u.ac.jp; 2 Graduate School of Biomedical and Health Sciences, Pharmaceutical Sciences, Hiroshima University, Hiroshima 739-8527, Japan; 3 Infectious Diseases Institute, The Ohio State University, Columbus, OH 43210, USA; 4 Center for Applied Plant Sciences, The Ohio State University, Columbus, OH 43210, USA

**Keywords:** lichen, *Penicillium*, fluorinated metabolite, antiproliferative

## Abstract

The U.S. endemic lichen (*Niebla homalea*)-derived *Penicillium aurantiacobrunneum* produced a cytotoxic paxisterol derivative named auransterol (**2**) and *epi*-citreoviridin (**6**). Feeding assay using ^13^C_1_-labelled sodium acetate not only produced C-13-labelled paxisterol but also confirmed the biosynthetic origin of the compound. The fluorination of bioactive compounds is known to improve pharmacological and pharmacokinetic effects. Our attempt to incorporate the fluorine atom in paxisterol and its derivatives using the fluorinated precursor sodium monofluoroacetate resulted in the isolation of 7-monofluoroacetyl paxisterol (**7**). The performed culture experiment, as well as the isolation and structure elucidation of the new fluorinated paxisterol, is discussed herein.

## 1. Introduction

**Figure 1:**
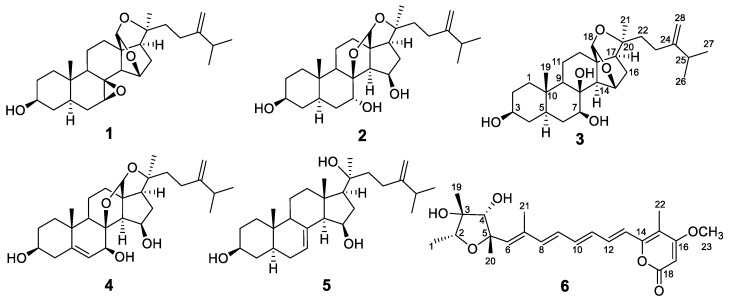
Structures of paxisterol (**1**), auransterol (**2**), (20*R*)-7,8-dihydroxypaxisterol (**3**), (15R*,20S*)-dihydroxyepisterol (**5**), and 4-epi-citreoviridin (**6**) isolated during the previous study [1].

In the continuation of our ongoing search of antiproliferative compounds from microbial associates of U.S. endemic lichens, we selected a bioactive fungus, *Penicillium aurantiacobrunneum* (Trichocomaceae)*,* which was isolated from *Niebla homalea* (Ramalinaceae), collected from coastal scrub with rock outcrops in Marin County, Point Reyes, California. Previous investigation on this strain cultured on brown rice led to the isolation of paxisterol (**1**) and its bioactive derivatives (**2**–**5**) together with *epi*-citreoviridin (**6**); see Figure 1 [1]. The antiproliferative mechanism of the most active paxisterol derivative, auransterol (**2**), was investigated and shown to inhibit cell proliferation by inducing apoptosis with a mechanism that is independent of the tumor suppressor p53. This was evidenced by the upregulation of apoptotic regulators such as BAX, cytochrome complex (Cyt-c), PARP-1, p21 and procaspase-3 proteins, and the downregulation of Bcl-2 with no modifications in procaspase-7 and p53 [2]. Paxisterol has been reported to be analgesic without anti-inflammation activity [3]. The bioactivity and druggability of molecules can be improved by incorporating appropriate pharmacophores and druggable chemical motifs into their structures [4]. Pharmacophores can be identified by evaluating the contribution of each functionality present in bioactive molecules to the activity via the evaluation of the activities of analogs [5,6]. With the aim to produce analogs for this purpose, the present study focuses on (**1**) feeding experiments to first confirm the biosynthetic origin of bioactive fungal paxisterols and (**2**) understanding if halogenated analogs can be produced by using halogenated derivatives of the identified precursor. Similar to previously published methods [7], we carried out a feeding assay using ^13^C-labelled glucose and identified pairs of directly coupled C-13-labelled carbon atoms incorporated in the biosynthesized paxisterol. The interpretation of the results concluded that two directly connected C-13 carbons of glucose were utilized during the biosynthesis. This finding was also confirmed by the feeding experiment using ^13^C-labelled sodium acetate, showing that acetyl coenzyme A was involved in paxisterol biosynthesis [8,9]. We thus hypothesized that fluorinated paxisterol can be produced if the biosynthetic enzyme that can use sodium monofluoroacetate is present in *P. aurantiacobrunneum*. The performed feeding experiments, as well as the isolation and structure elucidation of a new fluorinated paxisterol, are discussed herein.

## 2. Results

**Figure 2:**
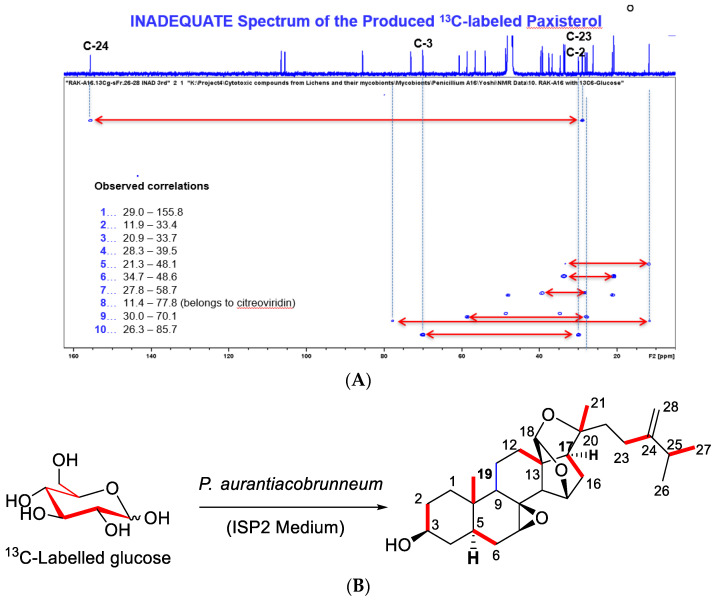
(**A**) Inadequate spectrum of ^13^C-labelled paxisterol (**1**); the one-dimensional ^13^C NMR of **1** was used for identification, red arrows are couples carbons; (**B**) structure of ^13^C-labelled paxisterol (**1**); red lines are identified ^13^C-^13^C bonds.

The biosynthesis of sterols has been studied using various methods, including C-13-labelled compounds, such as sodium acetate, leucine, mevalonic acid (MVA), and glucose. As a result, varieties of labelling patterns in the isoprenoid precursor, isopentenyl pyrophosphate (IPP), and sterols produced by the MVA and the non-mevalonate 2-C-methyl-d-erythritol 4-phosphate (MEP) pathways have been observed [7,9]. Focusing on our interest to bioengineer halogenated analogs of sterols using fungi such as the lichen-derived *Penicillium* species, we first performed feasibility studies using C-13-labelled sodium acetate and glucose as carbon source precursors. As a result, C-13-labelled paxisterol and epi-citreoviridin were produced. The labelling pattern of the produced compounds was analyzed using an inadequate nuclear magnetic resonance (NMR) experiment, since these compounds contain a high abundance of ^13^C-carbons. The connectivity of the ^13^C-labelled carbons in the paxisterol isolated during the feeding experiment is shown in Figure 2. While ^13^C incorporation was evidenced, we could observe non- or partially labelled paxisterols. These findings confirmed that our experimental setting will allow the production of paxisterol while using glucose or sodium acetate as precursors.

**Figure 3:**
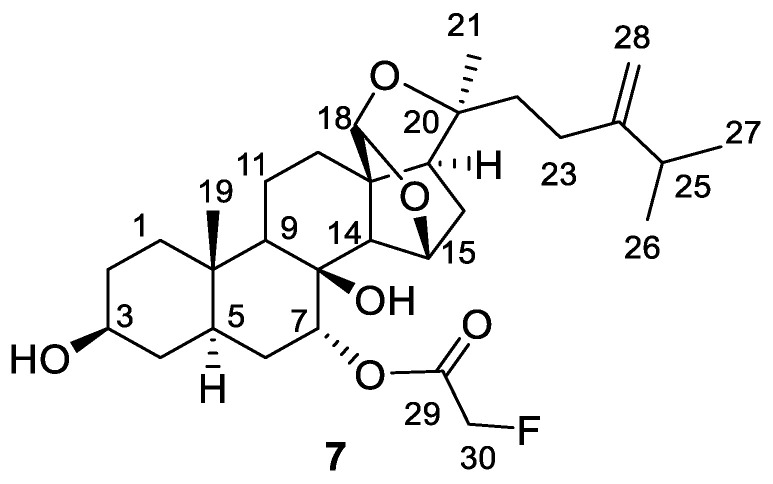
Structures of (20*R*)-7α-Fluoroacetoxy-8-hydroxypaxisterol (**7**).

Precursors are utilized by specific enzymes of the host to produce physiologically relevant compounds. We hypothesize that structurally similar precursors would afford similar outcomes. Next, we investigated this hypothesis by feeding a culture of *P. aurantiacobrunneum* with sodium monofluoroacetate to produce fluorinated paxisterol. Fluorine (^19^F) NMR experiments were performed on extracts, partitions and fractions in order to efficiently isolate only the produced fluorinated compounds. The ^19^F NMR spectra of prioritized extract and fractions containing fluorinated compounds are shown in Supplementary Materials (Figures S1 and S2). The identified fraction was subjected to flash column chromatography to yield compound **7**.

(20*R*)-7α-Fluoroacetoxy-8-hydroxypaxisterol (**7**, Figure 3) was isolated as a white amorphous solid. The molecular formula was established as C_30_H_45_FO_6_ from the observation of a sodiated molecular ion peak at 543.3101 (calculated for C_30_H_45_FO_6_Na^+^, 543.3092) in the high-resolution electrospray ionization mass spectrum (ESIMS), as well as other NMR spectroscopic data of **7**, such as ^13^C, HSQC and HMBC. The IR spectrum displayed stretching of an ester carbonyl and absorption band of hydroxyl functions. The ^1^H NMR spectroscopic data (Table 1) of **7** exhibited resonances of four methyl groups, two of which are singlets (*δ*_H_ 1.00 and *δ*_H_ 1.33, each 3H), while the remaining two are assignable to those of an isopropyl group (*δ*_H_ 1.03 (d, *J* = 6.8 Hz, 6H, and *δ*_H_ 2.26, sept, *J* = 6.8 Hz, 1H). In addition, there were three oxygen-bearing methines (*δ*_H_ 3.54, m, H-3, *δ*_H_ 4.30, brs, H-15, and *δ*_H_ 5.15, brt, *J* = 2.5 Hz, H-7); one acetal proton (*δ*_H_ 5.51, s, H-18); one exocyclic methylene group (*δ*_H_ 4.71 and *δ*_H_ 4.76, each brs, H-28a and H-28b); and one methylene group, which was coupled with fluorine (*δ*_H_ 4.96, dd, *J* = 46.8, 2.8 Hz, 2H). The ^13^C NMR spectrum together with data generated from HSQC and HMBC spectra indicated the presence of 31 carbon resonances, of which 28 were superimposable to (20*R*)-7,8-dihydroxypaxisterol (**3**), while the three remaining were assignable to those of monofluoroacetate (doublet at 77.8 ppm with a coupling constant *J* = 178.2 Hz, C-30 and a singlet carbon at 168.2 ppm, C-29). The presence of the monofluoroacetate in the molecule was confirmed by the observation of HSQC cross-peak between the doublet carbon signal of C-30 and the two doublets of the doublet proton signal at *δ*_H_ 4.96 (dd, *J* = 46.8, 17.6 Hz, H-30) of the fluoro-methylene and the HMBC correlation from H-30 to the ester carbonyl at 168.2 ppm.

**Figure 4:**
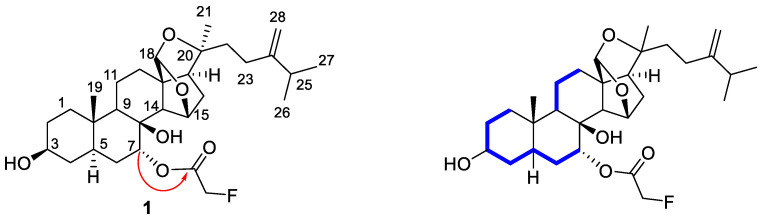
Key HMBC (red arrow) and TOCSY (blue line) correlations observed in compound **7**.

The attachment of the fluoroacetoxyl group to C-7 suggested by the presence of the downfield shift of the proton signal arising from H-7 (5.15 ppm vs. 3.74 ppm in **3**) was confirmed by the HMBC long-range correlation from H-7 (*δ*_H_ 5.15) to the ester carbonyl signal (C-29). Furthermore, the allocation of the methine carbinol at C-3 of the sterol skeleton and other functionalities, such as the acetal group at C-18 and the exocyclic methylene at C-24 of **7**, were confirmed by carrying out one- and two-dimensional NMR experiments. The selective irradiation of the oxygen-bearing methine signals at *δ*_H_ 3.54 (H-3) and *δ*_H_ 5.15 (H-7) of **7** using 1D TOCSY experiments identified proton spin networks from H-1 to H-7 (Figure 4). The acetal at C-18 was concluded by the HMBC cross-peaks from the acetal proton at 5.51 ppm and C-13, 15, 20, and C-14, while the exocyclic methylene was substantiated by the long-range correlation from the isopropyl methine (*δ*_H_ 2.26) to the methylene carbon at 105.8 ppm (C-28). Other important HMBC long-range and NOESY correlations that allowed us to elucidate and confirm the structure of **7** are shown in Figure 4. From the above data, the structure of **7** was deduced, as depicted.

Next, the ethyl acetate fraction was subjected to fluorine (^19^F) NMR experiments (^1^H coupled and decoupled experiments). As a result, the presence of fluorinated compounds was clearly observed in the two experiments performed (data not shown). However, fluorinated compounds were not isolated during this study due to their apparent instability during the isolation process.

Epoxide can open to become a dihydroxy group under basic conditions. We suspected that compound **7** can be abiotically produced in the presence of monofluoroacetate. It is worth noting that when pure paxisterol isolated from the same fungus was incubated with sodium monofluoroacetate in ISP2 medium without the fungus, compound **7** was not detected. This study concluded that compound **7** was only produced in the presence of *P. aurantiacobrunneum*.

To conclude, the presence of fluorinated compounds, as evidenced by the fluorine signals in ^19^F NMR experiments of the ethyl acetate fraction of the culture extract and the isolation of compound **7**, showed that halogenated precursors, such as fluorinated acetate, can produce fluorinated secondary metabolites. After the current feasibility study, we are focusing on scaling up cultures and isolating stable halogenated compounds.

## 3. Materials and Methods

^1^H and ^13^C NMR spectra were recorded at 25 °C with a Bruker Avance III 400 HD NMR spectrometer (Billerica, MA, USA) and Bruker Avance III HD Ascend 700 MHz (Billerica, MA, USA). High-resolution mass spectra were acquired with a Thermo LTQ Orbitrap (specifications: analyzers: ITMS and FTMS; mass range: 50–4000 *m*/*z*; resolution: 7500–100,000). Optical rotation was determined on an Anton Paar MCP 150 polarimeter.

### 3.1. Fungal Source

*Penicillium aurantiacobrunneum*, a lichen-associated fungus, which was used in this study, was isolated from the *Niebla homalea species* and identified as previously described [1]. A voucher specimen of this fungus was stored at −80 °C at the Division of Medicinal Chemistry and Pharmacognosy, College of Pharmacy, The Ohio State University, as RAK_A16.

### 3.2. Feeding Assays

#### 3.2.1. ^13^C-Labelled Glucose

A method similar to that reported in [7] was performed. Fungal colonies grown on ISP2 agar at 19 °C for 6 days was inoculated in ISP2 broth medium containing D-Glucose-^13^C_6_ (250 mg yeast extract, 625 mg malt extract, 250 mg D-Glucose-^13^C_6_, and 62.5 mL distilled water) and incubated at 20 °C, 150 rpm, for 14 days. After filtering the fungal culture with filter paper, an equal amount of 500 mL ethyl acetate was added to the liquid to extract the metabolites. The extraction process was repeated three times with equal amounts of ethyl acetate. The ethyl acetate fractions of the fungus were combined and dried under vacuum on Rotavap (~40 °C) to obtain 22.5 mg of residue containing ^13^C-labelled paxisterol (**1**).

#### 3.2.2. ^13^C-Labelled Sodium Acetate

Similar to the method described by Nebeta and coworkers [10], a seed of the fungal colony was cultivated into ISP2 broth medium containing sodium acetate-1-^13^C and incubated for 26 days at 20 °C (150 rpm). An equal amount of ethyl acetate was then added to the culture medium containing the culture, and after filtering through filter paper, the filtered mixture was extracted with 500 mL of ethyl acetate. The extraction process was repeated three times. The ethyl acetate fractions of the fungus were combined and dried under vacuum on Rotavap (~40 °C) to obtain 9.2 mg of residue containing ^13^C-labelled paxisterol (**1**).

### 3.3. Extraction and Isolation of Compound **7**

Fourteen-day fungal culture in ISP2 broth medium supplemented with sodium monofluoroacetate (1.0 g yeast extract, 2.5 g malt extract, 1.0 g sodium monofluoroacetate, and 250 mL distilled water) was extracted with (3 × 1 L) ethyl acetate at room temperature. The ethyl acetate fraction was evaporated under vacuum to yield 73.2 mg residue. The extract was then fractionated by C_18_ reversed-phase silica gel liquid chromatography using 40% aqueous methanol (50 mL), followed by 70% aqueous methanol (50 mL), and later washed with 100% methanol to yield three fractions (F1, 17.8 mg; F2, 15.1 mg; and F3, 17.3 mg). Fraction F3 was subjected to silica gel column chromatography eluted with a stepwise gradient of hexanes and ethyl acetate (from 100:0 to 0:100) to obtain ten sub-fractions (F3-1 through F3-10). Compound **7** (0.76 mg) was obtained from fraction F3-4.

(20*R*)-7α-Fluoroacetoxy-8-hydroxypaxisterol (**7**): white powder, [α]_D_ + 8 (c 0.05, MeOH); ^1^H NMR and ^13^C NMR spectral data (see Table 1); positive HRESIMS *m*/*z* 543.3101 ([M + Na]^+^, which corresponds to a molecular formula of C_30_H_45_FO_6_ (calcd. for C_30_H_45_FO_6_Na^+^, 543.3092).

## Figures and Tables

**Table 1:** ^1^H and ^13^C NMR NMR spectroscopic data for compounds **7** and **3**.

Position	(20*R*)-7α-Fluoroacetoxy-8-hydroxypaxisterol (7 ^a^)		(20*R*)-7,8-Dihydroxypaxisterol (3 ^b^)	
	^1^H	^13^C	^1^H	^13^C
	*δ*_H_ (m, *J* in Hz)	*δ*_C_, Type	*δ*_H_ (m, *J* in Hz)	δ_C_, Type
1	1.02 (m), 1.77 (m)	37.6, CH_2_	1.02 (m), 1.77 (m)	30.2, CH_2_
2	1.48 (m), 1.75 (m)	30.2, CH_2_	1.49 (m), 1.75 (m)	30.2, CH_2_
3	3.54 (m)	70.5, CH	3.58 (ddd, 15.8, 10.9, 4.7)	70.6, CH_2_
4	1.34 (m), 1.45 (m)	36.5, CH_2_	1.37 (m), 1.48 (dd, 12, 5.5)	36.7, CH_2_
5	1.57 (m)	37.5, CH	1.68 (m)	36.2, CH
6	1.34 (m), 2.09 (m)	29.3, CH_2_	1.26 (m), 2.06 (m)	32.1, CH_2_
7	5.15 (brt, 2.5)	74.7, CH	3.74 (t, 2.6)	71.4, CH
8		72.8, C		73.9, C
9	1.07 (m)	49.2, CH	1.11 (m)	48.1, CH
10		35.5, C		35.4, C
11	1.64 (m), 1.80 (m)	18.5, CH_2_	1.63 (m), 1.80 (m)	18.4, CH_2_
12	1.71 (m), 2.24 (m)	28.2, CH_2_	1.71 (m), 2.27 (m)	28.2, CH_2_
13		57.5, C		57.2, C
14	1.82 (m)	55.2, CH	2.23 (brs)	55.0, CH
15	4.30 (brs)	74.6, CH	4.34 (brs)	74.3, CH
16	1.70 (m), 1.92 (d, 13.1)	34.8, CH_2_	1.83 (m), 1.95 (m)	34.8, CH_2_
17	2.14 (d, 9.7)	49.2, CH	2.15 (d, 9.5)	49.1, CH
18	5.51 (s)	107.3, CH	5.54 (s)	107.3, CH
19	1.00 (a)	11.7, CH_3_	0.98 (s)	11.5, CH_3_
20		85.2, C		85.0, C
21	1.33 (s)	26.4, CH_3_	1.37 (s)	26.2, CH_3_
22 ^a,b^	1.75 (m)	39.8, CH_2_	1.79 (m)	39.6, CH_2_
23	1.95 (m), 2.02 (m)	29.5, CH_2_	2.01 (m)	29.1, CH_2_
24		155.8, C		155.7, C
25	2.26 (sept, 6.8)	33.9, CH	2.28 (sept, 6.8)	33.8, CH
26	1.03 (6.8)	21.1, CH_3_	1.06 (s)	20.9, CH_3_
27	1.03 (6.8)	21.1, CH_3_	1.06 (s)	20.9, CH_3_
28	4.71 (brs), 4.76 (brs)	105.8, CH_2_	4.73 (brs), 4.79 (brs)	105.6, CH_2_
CH_2_F	4.94 (dd, 46.8, 17.6), 4.97 (dd, 46.8, 17.6)	77.8 (178.2 Hz), CH_2_		
O-C=O		168.2, C		

^a^ 700 MHz for ^1^H NMR and 175 MHz for ^13^C, measured in CD_3_OD-*d*_4_, ^b^ from reference [1].

## Data Availability

Not applicable.

## Supplementary Materials

The following supporting information can be downloaded online. Figure S1: Summary of feeding assay; Figure S2: Fluorine NMR of the extracts and fractions; Figure S3: Mass spectrum of compound **7**.

## Sample Availability

Samples of the compounds are not available due to their scarcity and stability.
