# Supplementary material for: Understanding the Biosynthesis of Paxisterol in Lichen-Derived Penicillium aurantiacobrunneum for Production of Fluorinated Derivatives
Source: Molecules. 2022 Mar 2;27(5):1641. doi: 10.3390/molecules27051641 (PMC8911623; doi:10.3390/molecules27051641)
Supplement: Supplementary file 1 [file molecules-27-01641-s001.zip › molecules-1608844-supplementary.pdf]

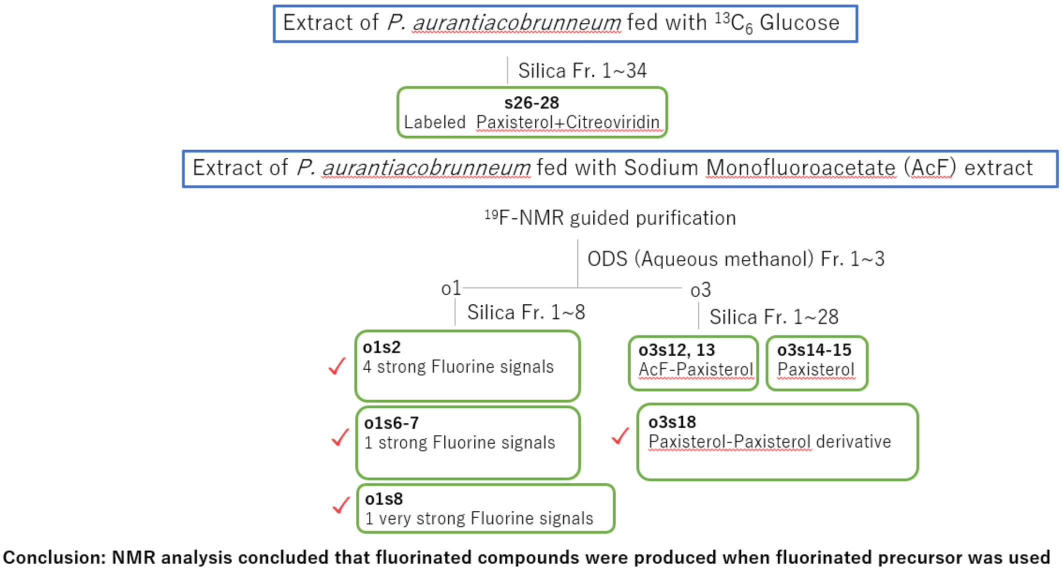

Figure S1. Summary of feeding assay.

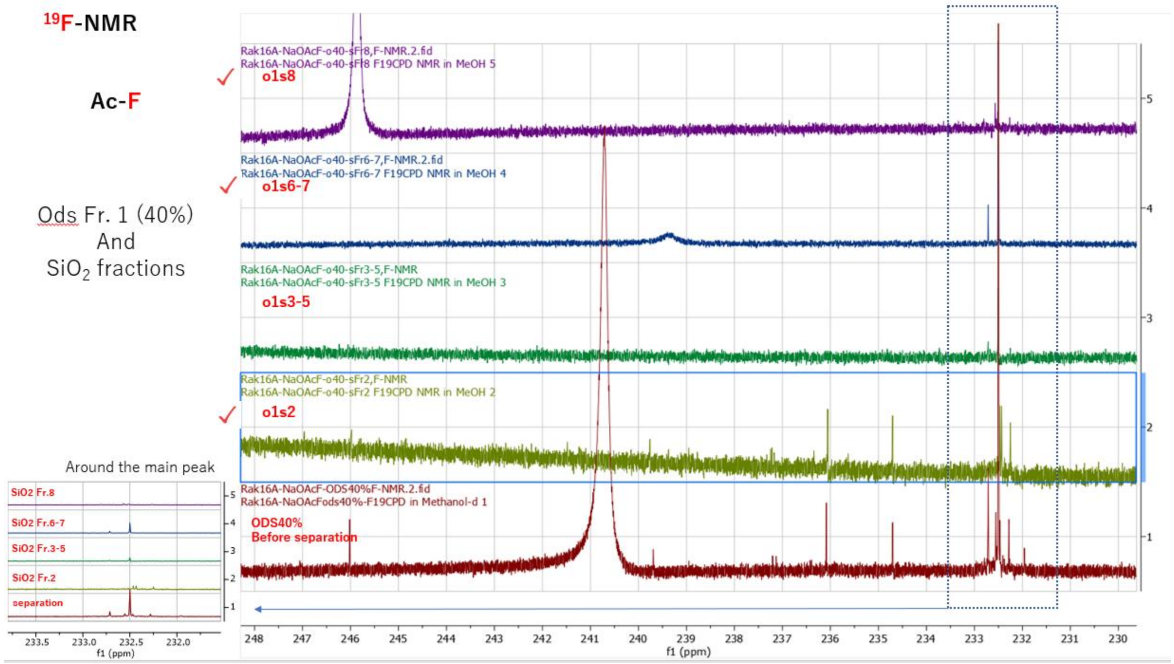

Figure S2. Fluorine NMR of the extracts and fractions.

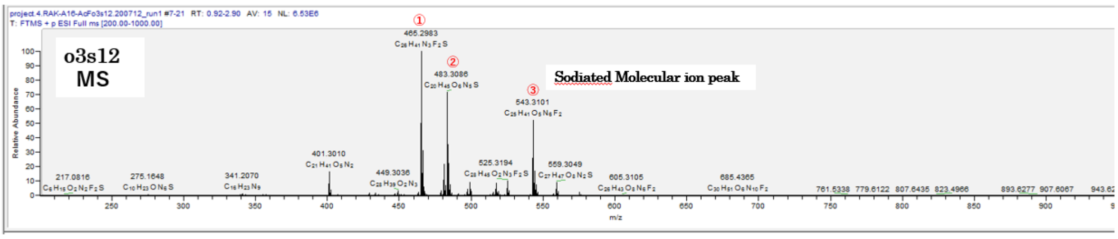

483.3086 – 465.2983 = 18.0103 (H_2O)

② - ①

543.3101-483.3086 = 60.0015

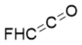

Chemical Formula: C_2HFO
Exact Mass: 60.0011

③ - ②

543.3101 – 465.2983 = 78.0118

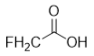

Chemical Formula: $C_2H_3FO_2$
Exact Mass: 78.0117

③ - ①

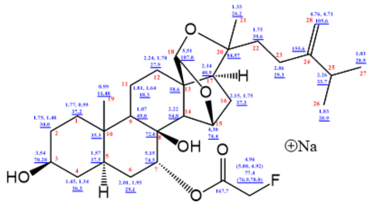

③: Sodiated molecular ion peak
Found *m/z* 543.3101
Calcd. for *m/z* 543.3092 $[C_{30}H_{45}FO_6Na]^+$

Figure S3. Mass spectrum of compound 7.
